# Community‐Based Approaches to Oral Frailty for Healthy Longevity in Japan: Bridging Screening, Community Action, and Integrated Care

**DOI:** 10.1111/ggi.70686

**Published:** 2026-08-02

**Authors:** Katsuya Iijima, Tomoki Tanaka, Kazunori Ikebe, Takayuki Ueda, Masanori Iwasaki, Tomoaki Mameno, Masahiro Akishita, Hidenori Arai, Hirohiko Hirano

**Affiliations:** ^1^ Institute of Gerontology The University of Tokyo Tokyo Japan; ^2^ Institute for Future Initiatives The University of Tokyo Tokyo Japan; ^3^ Department of Removable Prosthodontics and Gerodontology, Graduate School of Dentistry The University of Osaka Suita Osaka Japan; ^4^ Department of Removable Prosthodontics and Gerodontology Tokyo Dental College Tokyo Japan; ^5^ Division of Dental Public Health, Department of Oral Health Science, Graduate School of Dental Medicine Hokkaido University Sapporo Hokkaido Japan; ^6^ Tokyo Metropolitan Institute for Geriatrics and Gerontology Tokyo Japan; ^7^ National Center for Geriatrics and Gerontology Obu Aichi Japan

**Keywords:** aged, community health services, frailty, interprofessional relations, oral frailty

## Abstract

In super‐aged societies such as Japan, achieving “healthy longevity with well‐being” requires not only medical and long‐term care services but also a seamless continuum that integrates health promotion, frailty prevention, and community‐based support, along with age‐friendly physical and social environments that support functional ability, social participation, and independent living. Within this framework, oral frailty (OF)—defined as the accumulation of slight declines in oral function, including tooth loss, chewing and swallowing difficulties, oral dryness, and low articulatory oral motor skills—has emerged as a key indicator linking oral health to systemic frailty, disability, and mortality. Originating in Japan, the concept of OF emphasizes early detection and reversibility through multidisciplinary collaboration. The 2024 Consensus Statement issued by three academic societies (the Japan Geriatrics Society, the Japanese Society of Gerodontology, and the Japanese Association on Sarcopenia and Frailty) proposed a definition, conceptual model, and assessment using the Oral Frailty 5‐item Checklist (OF‐5). This review summarizes the development of OF initiatives within Japan's Community‐Based Integrated Care System and discusses recent international trends, including the WHO Global Oral Health Action Plan (2023–2030), the FDI policy statement “Oral Health for Healthy Ageing,” and emerging global research evidence. Practical examples, such as a community‐wide campaign in Hiratsuka City, illustrate multisectoral collaboration to prevent and raise awareness of OF. Finally, we highlight future directions, including integration of oral health into community development, strengthening interprofessional collaboration, and leveraging digital technologies for monitoring and education. By integrating clinical, community, and policy perspectives, the Japanese concept of OF offers a promising, implementable model for global healthy aging.

## Graphical abstract

To achieve healthy longevity, Japan has integrated the concept of “Oral Frailty (OF)” into its community care system to link oral health with individual/community well‐being. By utilizing the 2024 consensus framework and multisectoral collaboration, this approach offers a scalable, promising, implementable model for global healthy aging.

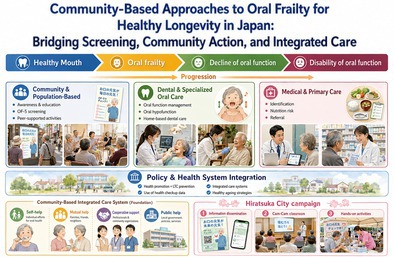

## 1 Introduction

Toward a Seamless Continuum for Healthy Longevity and Well‐Being: In this era, often referred to as the “100‐year lifespan era,” we have gained the ability to live longer lives. As longevity advances, conditions that cannot be completely cured are also increasing, specifically including dementia, locomotive syndrome, and frailty. Against this backdrop, it is urgent to pursue initiatives to achieve healthy longevity—preventing such conditions as much as possible, avoiding the need for long‐term care, and maintaining independent living for as long as possible. Furthermore, there is a strong demand for achieving longevity with well‐being, where individuals can eat independently until the end to maintain nutritional status and sustain social connections and interactions forever. Therefore, when aiming for these ideal goals, what should be communicated anew to the public, and which behavioral changes should be encouraged? Amidst a declining birthrate and aging population, two important perspectives exist. First, the perspective of health promotion and frailty prevention to achieve healthy longevity. Second, the perspective of multi‐layered care (medical‐nursing care coordination, interprofessional work) enables citizens to live out their lives in familiar surroundings. Crucially, these two perspectives must form a seamless, integrated continuum.

In this review, we aim to (1) summarize the development of the Japanese concept of oral frailty (OF) and its assessment framework, (2) describe how OF initiatives have been implemented within the Community‐Based Integrated Care System, (3) situate these efforts within recent international policies and emerging evidence, and (4) outline future directions for research and practice, including interprofessional collaboration and digital transformation.

## 2 The Concept of OF

In 2024, the Joint Working Group on Oral Frailty, established by the Japan Geriatrics Society, the Japanese Society of Gerodontology, and the Japanese Association on Sarcopenia and Frailty, published a consensus statement to promote public understanding of OF [1]. This initiative responded to trends highlighted in the Japanese Association for Dental Science conference report, which defined “oral health management” and “OF.” Importantly, the report distinguishes between natural, age‐related decline in oral function, which is inevitable, and OF, which is multifactorial and potentially reversible. OF involves declines in oral function due to physical, social, mental, and cognitive factors. Thus, while aging is progressive and unavoidable, OF represents a condition with various treatment possibilities. Early identification and intervention can mitigate or even reverse its trajectory.

The aim of this consensus statement is to prevent and mitigate OF by enhancing multidisciplinary collaboration. OF, marked by slight declines in functions such as tooth loss, can lead to physical frailty, sarcopenia, and malnutrition. Because co‐occurrence with frailty and comorbidities elevates risks of disability and death, the statement proposes a unified definition of OF.

### 2.1 The Concept of OF

*OF is a state of oral function between the normal state of a “healthy mouth” and the “decline of oral function.”*

### 2.2 The Definition of OF

*OF is characterized by the accumulation of slight declines in oral function, including tooth loss and difficulties in eating and communicating, which increases the risk of impaired oral functional capacity. However, proper intervention and treatment can effectively improve this condition*.

The intention of this consensus statement was to raise public awareness of early, slight declines in oral function and to introduce OF as a new concept grounded in the multifaceted frailty model. Two conceptual diagrams were developed: one for healthcare professionals and one for the general public. Both illustrate the continuum from healthy oral function to OF, frailty, sarcopenia, and malnutrition. The professional version emphasizes the Oral Frailty 5‐item Checklist (OF‐5), while the public version simplifies the framework for general understanding.

Assessment of OF is supported by the OF‐5, a practical screening tool developed from validated items. OF is defined as meeting at least two of five criteria: fewer teeth, chewing difficulty, swallowing difficulty, dry mouth, and low articulatory oral motor skills. Notably, the checklist can be used outside of dental settings, making it accessible in community and healthcare contexts. Official translations of OF‐5 are being prepared in multiple languages, with a view toward global application. In conclusion, the Joint Working Group highlights OF as a reversible, multifactorial condition distinct from natural aging. Through conceptual clarification, visual frameworks, and the OF‐5, the group seeks to advance early detection, public awareness, and preventive strategies, thereby improving nutrition, functional health, and longevity outcomes in older adults.

### 2.3 Approaches to OF

**FIGURE 1:**
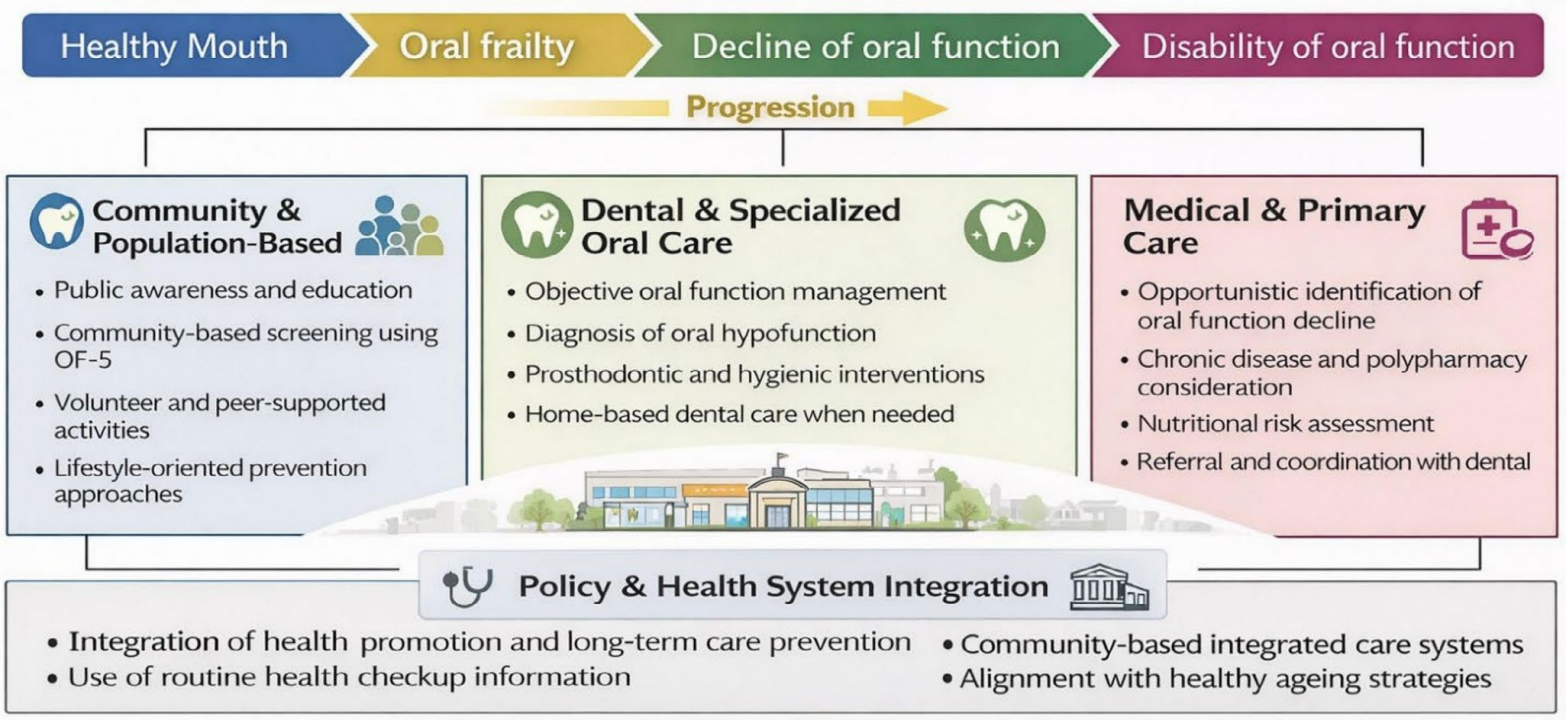
Operationalization of the oral frailty (OF) concept across community, clinical, and policy settings. This figure illustrates the conceptual continuum of oral health from a healthy mouth to OF, decline of oral function, and disability, and maps corresponding actions across community and population‐based prevention, dental and specialized oral care, medical and primary care, and policy and health system integration. The framework emphasizes OF as a potentially reversible stage and highlights the importance of coordinated, multidisciplinary approaches across settings. OF‐5, Oral Frailty 5‐item Checklist.

It has been clearly indicated that OF requires a comprehensive approach through collaboration among healthcare professionals (Figure 1). The type of intervention varies according to the degree of oral function decline, ranging from population approaches to high‐risk approaches. In the early stages, population‐wide interventions such as improvements in lifestyle habits and the promotion of oral health awareness were effective. In contrast, once functional decline becomes apparent, strategies focusing on the early identification of at‐risk individuals and targeted interventions are necessary. These approaches are complementary, and effective implementation requires multidisciplinary collaboration.

During the stage of “decline of oral function,” oral hypofunction plays a key role as a dental‐specific clinical entity [2]. Oral hypofunction is a diagnostic concept established when dentists diagnose based on objective oral function tests and when dentists and dental hygienists provide oral function management.

Although OF, which is assessed primarily through subjective evaluation, and oral hypofunction, which is diagnosed based on objective assessments using testing devices, overlap in many respects, they are not in a simple hierarchical relationship. Rather, oral hypofunction can be regarded as a specific clinical intervention within the broader concept of OF, corresponding to a high‐risk approach. In other words, while OF represents a comprehensive framework that should be addressed through public awareness and multidisciplinary collaboration, oral hypofunction and its associated oral function management provide a practical means of intervention primarily carried out by dentists and dental hygienists. Thus, OF and oral hypofunction are closely related yet distinct concepts, and organizing interventions around their proper positioning will contribute to the maintenance of oral function and the promotion of systemic health in older adults.

## 3 Expanding the Role of Healthcare Professionals in Oral Function Maintenance

Numerous specialized professionals in the medical and nursing care fields can advance various clinical activities in ways specific to their individual domains. However, whether it is the early prevention phase centered on a population approach or the high‐risk approach phase where eating and swallowing dysfunction is progressing, residents and patients should be approached under multidisciplinary collaboration involving multiple professionals. All professionals are present at front touchpoints with residents, and this concept of OF should be emphasized by them.

Oral functions, of course, have the primary role of “chewing food thoroughly,” but it also plays a significant role in social aspects such as “tasting and enjoying,” and “speaking and laughing as one of multi‐faceted sociality.” Reflecting on past clinical settings and various activities in community settings, other professions, including physicians, often lacked a strong awareness of their own roles. Rather than having only professionals in dental field (e.g., dentists, dental hygienists) advocate/contribute for the diverse oral functions, all other professionals (e.g., physicians, pharmacists, nurses, physical therapists, occupational therapists, speech therapists, dietitians, care managers, care staff, etc.) should engage in raising awareness about the importance of oral functions, including OF. This will clearly accelerate changes in residents' awareness and behaviors toward maintaining oral function.

Specifically, many patients seen by primary care physicians (those with chronic diseases or multiple conditions) may also have minor declines in oral function. We strongly hope physicians and pharmacists dispensing prescriptions to actively educate these individuals about OF. Simultaneously, they should encourage these patients to establish a regular dental care provider and attend regular maintenance appointments. Other professionals who interact with residents in diverse settings should also emphasize the importance of oral function—not only for maintaining nutritional management but also for social well‐being—from various professional perspectives.

**FIGURE 2:**
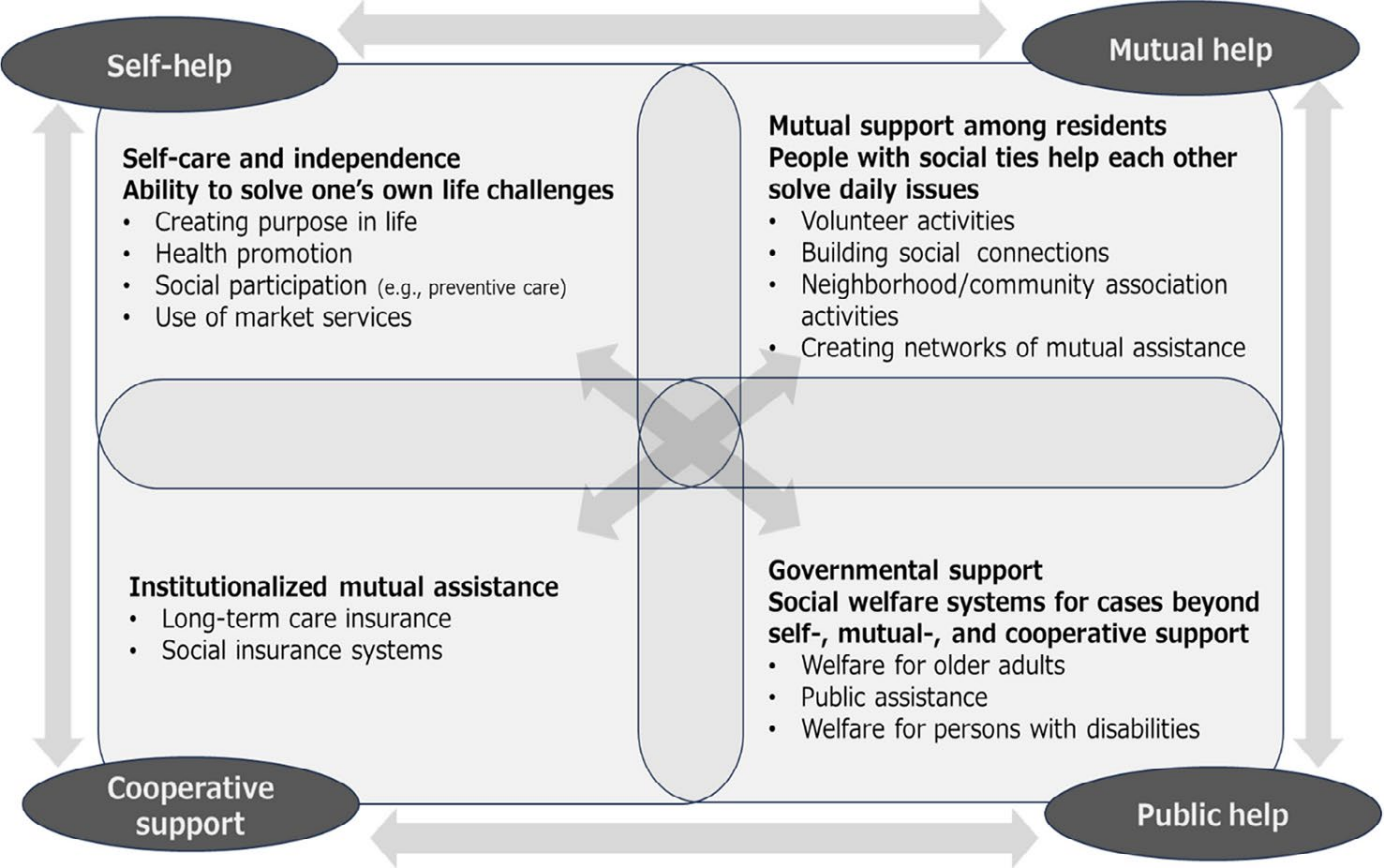
Conceptual framework of a Community‐Based Integrated Care System. This figure illustrates the foundational structure of a Community‐Based Integrated Care System, which is composed of four interrelated domains: Self‐help, mutual help among residents, cooperative support, and public help. Self‐help emphasizes individual self‐care, independence, and health‐promoting behaviors. Mutual help represents informal social support and reciprocal assistance within communities. Cooperative support refers to organized and institutionalized forms of mutual assistance, including social insurance systems. Public help encompasses formal governmental and social welfare services for situations that cannot be sufficiently addressed through self‐help, mutual help, or cooperative support. These domains interact dynamically and complement each other to support daily living, well‐being, and care needs within the community.

In Japan, the development of a Community‐Based Integrated Care System (CBICS) has been promoted as a foundation that enables older adults to live with dignity and independence in their familiar communities. The CBICS is designed to provide an integrated system of medical care, long‐term care, preventive services, housing, and daily life support, thereby allowing individuals who require care to continue their preferred lifestyles at home for as long as possible. Its operational concept is based on the principles of self‐help, mutual aid among residents, community‐based mutual support, and public support, as illustrated in Figure 2.

Oral health programs for older adults must be implemented in accordance with this framework, with its holistic and integrative nature taken into account. It is therefore essential that community residents and professionals in medicine, nursing, and welfare share a common understanding of the importance of maintaining, improving, and restoring oral function as a means of extending healthy life expectancy.

Within the CBICS, local dental associations play a key professional role in promoting OF prevention and management. In addition to their responsibilities for improving the quality of dental care and advancing community oral health, dental associations collaborate closely with local governments to promote oral health programs for older adults. The Japan Dental Association disseminates scientific and public information on OF [3, 4], and based on these resources, local dental associations have been actively engaged in implementing OF countermeasures within the CBICS framework.

Such community‐based OF initiatives combine population approaches—such as health education and public awareness campaigns—with high‐risk approaches, including individualized instruction and outreach for persons with declining oral function, as well as clinical approaches such as home‐visit dentistry for those requiring home care. These efforts are tailored to local characteristics and residents' needs and are implemented in close collaboration with municipal administrations.

The 2024 Joint Statement issued by three academic societies clarified the conceptual relationships among OF, oral hypofunction, and dysphagia [1, 2]. This statement has facilitated the development of oral health programs that are appropriately timed and adapted to the oral and systemic conditions of community‐dwelling older adults, in alignment with the philosophy of the CBICS. Such an integrated approach is pivotal and may also provide valuable insights for countries with differing healthcare and welfare infrastructures when developing oral health initiatives for older adults.

## 4 Trends in Domestic and International Evidence on OF

OF is a concept first proposed in Japan in 2014. It refers to the accumulation of multiple mild declines in oral function—such as tooth loss, chewing difficulty, swallowing difficulty, oral dryness, and low articulatory oral motor skills—and has been shown in longitudinal studies to be significantly associated with adverse outcomes, including physical frailty, sarcopenia, disability, and mortality [5].

The background to the emergence of this concept lies in the recognition that, with population aging, traditional morphological assessments such as “number of teeth” or “occlusal status” were insufficient to explain functional capacity and healthy life expectancy in older adults in Japan. Although previous studies had suggested that deterioration in chewing, swallowing, and articulation could lead to undernutrition and physical frailty, no index existed to comprehensively evaluate these functions [6, 7].

The initial concept of OF defined it as a state in which multiple mild declines in oral function, occurring with aging, interact to affect daily functioning and health. It focuses not on single‐function impairments but on complex, multidimensional functional decline, emphasizing the importance of early detection at a subclinical stage. This interdisciplinary preventive approach stresses the possibility of intervention while symptoms remain mild, and presupposes collaboration among multiple professionals, including dentists, physicians, dietitians, nurses, care workers, and pharmacists.

In Japan, the predictive validity of OF has been verified in epidemiological studies among community‐dwelling older adults [8, 9, 10, 11]. The recently developed OF‐5 is a concise and accessible screening tool usable even by non‐dental professionals [12, 13]. The OF‐5 consists of five components: “fewer teeth,” “difficulty in chewing,” “difficulty in swallowing,” “dry mouth,” and “low articulatory oral motor skills.” If two or more of these five criteria are met, the individual is classified as having OF. Studies using the OF‐5 have reported that approximately 40% of community‐dwelling older adults meet the criteria for OF, which is associated with physical frailty, low dietary variety, social isolation, decreased psychological well‐being, depressive mood, increased risk of mild cognitive impairment, disability, and mortality [12, 13, 14, 15, 16]. Furthermore, in April 2024, the “Joint Working Committee on Oral Frailty” by three academic societies: the Japan Geriatrics Society, the Japanese Society of Gerodontology, and the Japanese Association on Sarcopenia and Frailty, issued Consensus Statement on “OF,” clarifying the conceptual definition, assessment methods, and preventive strategies for OF, emphasizing the need for multidisciplinary collaboration and presenting concept diagrams for both the general public and professionals [1].

Internationally, movements related to the OF concept are also gaining momentum. The World Health Organization (WHO), in its *Global Oral Health Action Plan 2023–2030*, defines oral health as: “Oral health is the state of the mouth, teeth and orofacial structures that enables individuals to perform essential functions, such as eating, breathing and speaking, and encompasses psychosocial dimensions, such as self‐confidence, well‐being and the ability to socialize and work without pain, discomfort and embarrassment [17].” This definition views oral health as part of functional capacity, encompassing not only basic functions such as eating, speaking, and breathing but also the maintenance of psychosocial aspects. It explicitly states that maintaining oral function is indispensable for achieving healthy aging—an approach that aligns with OF's advocacy for a shift toward a functional perspective.

The FDI “Oral health for healthy ageing” policy statement is an international policy document developed in response to global population aging and the need to extend healthy life expectancy [18]. First adopted at the 2015 FDI General Assembly, it was revised in 2023 to align with the WHO's *Decade of Healthy* Aging *(2021–2030)* and *Global Oral Health Action Plan 2023–2030* [18, 19]. The revised version defines healthy aging as “reacquiring and maintaining the functional capacity that promotes well‐being as people age,” and clearly states that maintaining and promoting oral functions—such as eating, speaking, and social interaction—contributes to delaying or preventing frailty. It also emphasizes that oral health care for older adults should be integrated into primary and general health services, and that this requires multidisciplinary collaboration. This policy vision is consistent with the OF concept in its focus on functional perspectives, explicit linkage to frailty prevention, and promotion of multidisciplinary and integrated approaches. However, there are differences in scope and standpoint: OF is a clinical and epidemiological model accompanied by an assessment tool (e.g., OF‐5) and is mainly applied for interventions and evaluation at the individual or community level, whereas the FDI statement is a policy document aimed at guiding health system development at national or regional levels and does not specify particular assessment tools. Nevertheless, the two are complementary. Combining the FDI's global policy vision with the concrete assessment framework and epidemiological evidence of OF could facilitate the international deployment of a functional oral health model that enables intervention from an early stage, thereby contributing to extending healthy life expectancy.

In Europe, a 2024 e‐Delphi study involving multidisciplinary experts reached strong consensus on eight core components for an operational definition of OF: (1) difficulty eating hard or tough foods; (2) inability to chew all types of foods; (3) decreased ability to swallow solid foods; (4) decreased ability to swallow liquids; (5) overall poor swallowing function; (6) impaired tongue movement; (7) speech or phonatory disorders; and (8) hyposalivation or xerostomia [20]. In Asia, the Chinese version of the OF‐5 has been translated and content validated [21]. Furthermore, a 2024 systematic review and meta‐analysis (*n* = 17 studies; total 24 983 participants) found that the overall prevalence of OF among older adults—predominantly in Asia—was 28% (95% confidence interval: 20%–36%), with differences depending on country and assessment tool. Major associated factors included age, sex, physical frailty, living situation, income, polypharmacy, and systemic diseases such as depression and diabetes [22].

In summary, the Japan‐originated OF concept is becoming well established domestically, supported by well‐designed epidemiological evidence and policy implementation. Internationally, challenges remain in standardizing definitions and assessment methods, ensuring cultural adaptability, and accumulating longitudinal data. Moving forward, it will be necessary to adapt Japanese knowledge to the social and cultural contexts of each country, and to build a global evidence base for OF prevention through international comparative studies and multi‐regional intervention research.

## 5 Future Directions and Issues

### 5.1 Strengthening Multisectoral Collaboration and Integrating Community Development

Addressing OF as part of comprehensive frailty prevention may require moving beyond discipline‐specific interventions toward integrated, multisectoral collaboration. OF is not only a dental or medical issue but is likely to relate to nutrition, mobility, social participation, and psychological well‐being [1]. Therefore, effective prevention may benefit from the involvement of diverse stakeholders—including government agencies, healthcare and welfare professionals, academic institutions, industry partners, and community organizations. Strengthening such collaboration could help embed OF prevention into community development, making initiatives more sustainable and responsive to local contexts.

The integration of oral health into community development appears particularly important. When oral health promotion is combined with initiatives such as social participation programs, nutritional support, and age‐friendly urban design, synergistic benefits may be generated that extend beyond clinical outcomes. Older adults may also be more likely to engage in preventive behaviors if oral health is perceived as part of everyday community life, rather than as a separate clinical issue.

Nevertheless, several challenges can be anticipated. First, coordination across different sectors may require new governance mechanisms, as existing systems often remain siloed. Second, a balance may need to be struck between professional‐led initiatives and citizen empowerment to avoid overly top‐down approaches. Third, ensuring equity is essential, since interventions may not adequately reach vulnerable groups with low health literacy, financial barriers, or social isolation. Fourth, within communities, it is important to improve the understanding of oral function decline among dental professionals and to build clinical systems that can respond appropriately. In parallel, fostering mutual understanding among different professional groups and creating opportunities for interprofessional collaboration is also crucial. Finally, evidence regarding cost‐effectiveness and long‐term outcomes remains limited, which could pose challenges for policymakers in allocating resources.

In summary, strengthening multisectoral collaboration and integrating oral health into community development could represent promising strategies for advancing frailty prevention. These approaches may offer a framework for sustainable, inclusive, and population‐level interventions, with potential applicability to other super‐aged societies worldwide.

### 5.2 Multisectoral Collaboration: A Local Model of Community‐Oriented OF Prevention

**FIGURE 3:**
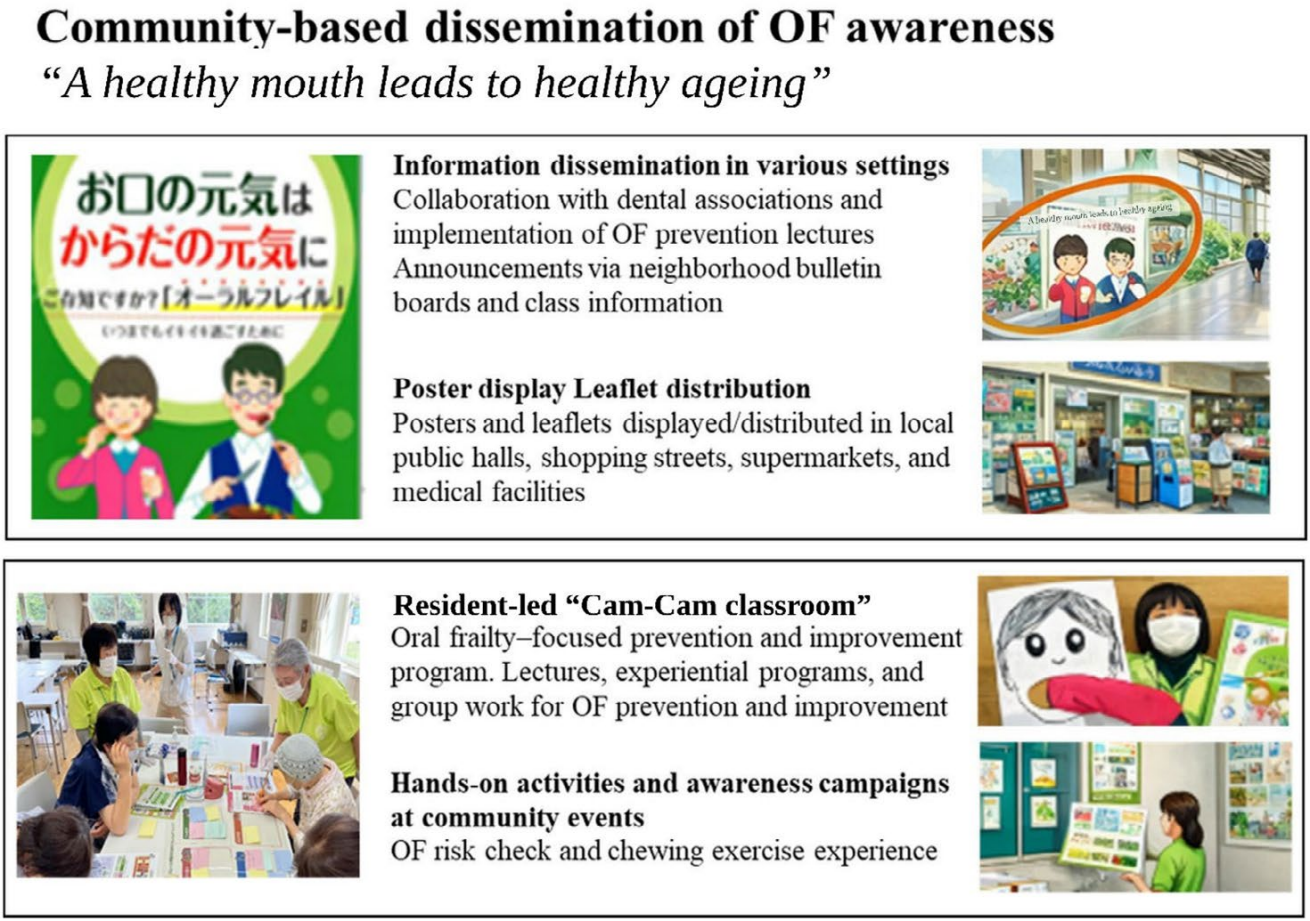
Community‐Based Multi‐Channel Dissemination and Oral Frailty Prevention Activities. This figure illustrates the multi‐channel, community‐based dissemination strategy and intervention activities implemented to promote awareness and prevention of OF. The campaign “A healthy mouth leads to healthy ageing” was promoted through multiple everyday settings, including dental clinics, shopping streets, supermarkets, apartment entrances, public halls, and train stations. Information was disseminated through poster displays in daily‐life settings, including apartment entrances, shopping streets, supermarkets, drugstores, dental clinics, and railway stations (display period: February–September 2023), as well as leaflet distribution (approximately 2300 copies). In addition, community outreach activities were conducted at local public events held at community centers, where oral function checks and chewing‐related experiential activities were provided. Furthermore, resident‐led “Cam‐Cam classroom (oral frailty–focused prevention and improvement program)” was implemented through 10 sessions. These activities were conducted in collaboration with local dentist associations, municipal governments, social welfare councils, and trained frailty supporters, aiming to integrate OF prevention into routine community environments. OF, oral frailty.

Hiratsuka City in Kanagawa Prefecture provides a notable example of a multisectoral, community‐wide approach to prevent OF as part of broader frailty prevention (Figure 3). Since 2022, the city has implemented an “OF Community Awareness Campaign,” mobilizing expertise from university researchers, local government, industry, and community groups to deliver education and screening in selected neighborhoods. Central to this initiative were “frailty supporters of volunteer‐driven frailty checkups program,” trained local volunteers who conducted prevention classes and outreach activities [23, 24].

The campaign combined oral function questionnaires with educational workshops, resulting in significant increases in awareness. Recognition of OF among participants increased from 59.8% to 75.5%. In addition, the initiative has been perceived as contributing to healthier lifestyles and may help delay or mitigate functional decline at the population level, potentially reducing the burden on long‐term care systems in the future.

The Hiratsuka model demonstrates several important strengths. Awareness‐raising activities were implemented not only in medical institutions and public facilities but also in shopping streets and through neighborhood circulars, ensuring that information reached residents in familiar community settings. The program benefited from a well‐established base of volunteers who had already been involved in frailty prevention and frailty check programs. Many of these individuals further enhanced their expertise by completing Kanagawa Prefecture's “OF Promotion Leader” training course, enabling them to play a more active role in delivering oral health initiatives. Local dental professionals, including dentists and dental hygienists, also contributed by providing professional consultation and follow‐up, thereby strengthening the clinical dimension of the program. Importantly, a social marketing approach was adopted: residents co‐developed the slogan “Oral vitality leads to overall vitality,” which spread organically through community‐driven channels and reinforced both awareness and ownership of the initiative. Finally, the program received policy‐level recognition; in 2025, Hiratsuka was the only municipality in Kanagawa to be awarded a “Performance‐based insurer function grant,” underscoring its value as a model of community‐based preventive care.

Challenges remain for scaling this model elsewhere, including sustaining volunteer engagement, ensuring equitable access across diverse communities, and securing stable funding. Nonetheless, Hiratsuka's pioneering case illustrates how multisectoral collaboration anchored in community structures can foster OF prevention, enhance awareness, and strengthen the foundation for sustainable healthy aging.

### 5.3 Integrated Implementation of Health Services and Long‐Term Care in Japan and OF

The integrated implementation of preventive health services and long‐term care prevention in Japan aims to extend healthy life expectancy and support independent living among older adults. Established through the 2020 legal reform, this framework targets those aged 75 and older, assessing lifestyle and health status while providing tailored advice. Municipalities take the lead in program management, evaluation, and individualized support, including encouragement to join community activities.

Within this framework, the Questionnaire for Medical Checkup of Old‐Old used to assess daily functioning includes two items on oral function [25]. These overlap partly with the OF‐5, and when combined with claims data, they allow municipalities to identify older adults at risk of oral function decline and designate them as program participants. These two oral function items alone have been suggested to be associated with higher long‐term care expenditures [26, 27]. Further, overlapping poor results on other questionnaire items and the coexistence of chronic diseases appear to further increase the risk of long‐term care need [27].

Such integration positions oral health as a core component of comprehensive frailty prevention alongside nutrition, physical activity, cognition, and social participation, and is equally important as strategies addressing physical frailty, chronic disease management, and individuals with unknown health status.

### 5.4 Digital Transformation in OF Prevention: Current Trends and Future Directions

Recent developments in digital health interventions for preventing sarcopenia and frailty have shown promising results—particularly in enhancing physical activity, balance, muscle strength, and quality of life among at‐risk older adults, as evidenced by systematic reviews and meta‐analyses published in 2025 [28, 29]. Although current guidelines underscore that existing evidence remains limited, digital modalities such as online exercise programs, mobile applications for activity tracking, and wearable devices are emerging as key tools for lifestyle‐based interventions.

For OF, these technologies hold significant potential. Wearable sensors could monitor chewing patterns or swallowing efficiency, while apps might deliver self‐administered OF‐5 screening or personalized oral function education. Integrating AI‐driven speech analysis or remote coaching via telehealth may facilitate early detection of oral decline and support exercise‐based preventive interventions. However, translating these innovations to real‐world oral health practice requires careful validation. Future research should prioritize robust evidence generation—leveraging patient‐reported outcomes, quality‐of‐life metrics, real‐world data, and cost‐effectiveness studies. Ensuring usability for older adults—through intuitive interfaces and adaptive design—is also critical.

In summary, although digital health research in frailty has not yet fully encompassed oral function, the current momentum and emerging technologies offer a fertile ground for future innovation in OF prevention—advancing toward scalable, data‐driven, and user‐centered strategies.

## 6 Summary

To achieve healthy longevity through frailty prevention, three pillars (three key points) are essential: (1) Nutrition (diet and oral function), (2) Physical activity (exercise and daily living activities), and (3) Social participation. It is crucial to recognize that connections and interactions within local communities are also highly important. Furthermore, early detection at the OF stage, self‐awareness, and subsequent behavioral changes, and early intervention hold significant importance. They are highly anticipated as a strategy to break the negative spiral (negative chain) of frailty at an earlier stage.

The number of academic articles related to oral function decline (oral dysfunction), including OF, has increased significantly. However, looking back at history, the evaluation methods, approaches to residents, and techniques for behavioral change have not yet reached a fully established stage. Given this background, the release of a joint statement on OF by three Japanese academic societies on April 1, 2024, and the accompanying introduction of a simplified screening index (OF‐5) are of significant importance. We anticipate that the statement's content, including this simple OF‐5 index, will be widely utilized, accelerating public awareness and education regarding the maintenance of oral function. Simultaneously, we strongly hope to see the development of healthcare systems capable of effectively supporting public behavioral change. This includes comprehensive oral function assessment and holistic guidance within the dental field, as well as multifaceted information provision, guidance, and referrals to dental care from all non‐dental professionals, thereby promoting interprofessional collaboration. Furthermore, we aim to disseminate this concept of OF, born in Japan, not only domestically but also internationally.

Finally, the time has come to reexamine and reevaluate conventional (existing) health promotion and preventive care initiatives and introduce fresh perspectives. For our nation to enter a new stage, the key will be how effectively and sustainably we achieve a “comprehensive community‐wide approach,” centered on administrative reform and fully grounded in both new and existing evidence.

## Funding

This work was supported by SUNSTAR Inc. Joint research funds and LOTTE Co. Ltd. Joint research funds.

## Data Availability

The data that support the findings of this study are available from the corresponding author upon reasonable request.

## References

[1] T. Tanaka , H. Hirano , K. Ikebe , et al., “Consensus Statement on “Oral Frailty” From the Japan Geriatrics Society, the Japanese Society of Gerodontology, and the Japanese Association on Sarcopenia and Frailty,” Geriatrics & Gerontology International 24, no. 11 (2024): 1111–1119. 39375858 10.1111/ggi.14980 PMC11843523

[2] S. Minakuchi , K. Tsuga , K. Ikebe , et al., “Oral Hypofunction in the Older Population: Position Paper of the Japanese Society of Gerodontology in 2016,” Gerodontology 35, no. 4 (2018): 317–324. 29882364 10.1111/ger.12347

[3] Japan Dental Association , “Manual on Implementing Oral Frailty Countermeasures in Dental Clinics,” 2019, https://www.jda.or.jp/en/pdf/Oral_Frailty_Manual‐Whole_Version.pdf?20230620.

[4] “Manual on Implementing Oral Frailty Countermeasures in Community Settings: Towards Integrated Implementation of Healthcare Services and Long‐Term Care Need Prevention for Older Adults 2020 Edition. Summary Leaflet,” 2020, https://www.jda.or.jp/oral_frail/2020/pdf/2020‐manual‐all.pdf.

[5] T. Tanaka , K. Takahashi , H. Hirano , et al., “Oral Frailty as a Risk Factor for Physical Frailty and Mortality in Community‐Dwelling Elderly,” Journals of Gerontology. Series A, Biological Sciences and Medical Sciences 73, no. 12 (2018): 1661–1667. 29161342 10.1093/gerona/glx225

[6] C. Inomata , K. Ikebe , R. Kagawa , et al., “Significance of Occlusal Force for Dietary Fibre and Vitamin Intakes in Independently Living 70‐Year‐Old Japanese: From SONIC Study,” Journal of Dentistry 42, no. 5 (2014): 556–564. 24589846 10.1016/j.jdent.2014.02.015

[7] Y. Watanabe , H. Hirano , H. Arai , et al., “Relationship Between Frailty and Oral Function in Community‐Dwelling Elderly Adults,” Journal of the American Geriatrics Society 65, no. 1 (2017): 66–76. 27655106 10.1111/jgs.14355

[8] D. Hoshino , H. Hirano , A. Edahiro , et al., “Association Between Oral Frailty and Dietary Variety Among Community‐Dwelling Older Persons: A Cross‐Sectional Study,” Journal of Nutrition, Health & Aging 25, no. 3 (2021): 361–368. 10.1007/s12603-020-1538-6 PMC12280587 33575729

[9] M. Iwasaki , Y. Watanabe , K. Motokawa , et al., “Oral Frailty and Gait Performance in Community‐Dwelling Older Adults: Findings From the Takashimadaira Study,” Journal of Prosthodontic Research 65, no. 4 (2021): 467–473. 33612666 10.2186/jpr.JPR_D_20_00129

[10] M. Nagatani , T. Tanaka , B. K. Son , et al., “Oral Frailty as a Risk Factor for Mild Cognitive Impairment in Community‐Dwelling Older Adults: Kashiwa Study,” Experimental Gerontology 172 (2023): 112075. 36581224 10.1016/j.exger.2022.112075

[11] M. Nishimoto , T. Tanaka , H. Hirano , et al., “Severe Periodontitis Increases the Risk of Oral Frailty: A Six‐Year Follow‐Up Study From Kashiwa Cohort Study,” Geriatrics 8, no. 1 (2023): 25. 36826367 10.3390/geriatrics8010025 PMC9956982

[12] M. Iwasaki , M. Shirobe , K. Motokawa , et al., “Prevalence of Oral Frailty and Its Association With Dietary Variety, Social Engagement, and Physical Frailty: Results From the Oral Frailty 5‐Item Checklist,” Geriatrics & Gerontology International 24, no. 4 (2024): 371–377. 38390632 10.1111/ggi.14846

[13] T. Tanaka , H. Hirano , K. Ikebe , et al., “Oral Frailty Five‐Item Checklist to Predict Adverse Health Outcomes in Community‐Dwelling Older Adults: A Kashiwa Cohort Study,” Geriatrics & Gerontology International 23, no. 9 (2023): 651–659. 37661091 10.1111/ggi.14634 PMC11503571

[14] K. Kawamura , K. Maeda , S. Miyahara , et al., “Relationship Between Depressive Moods and Oral Frailty in a Frailty Outpatient Clinic: A Cross‐Sectional Study,” Gerontology 72, no. 3 (2026): 202–210. 41397001 10.1159/000549877

[15] T. Tanaka , W. Lyu , H. Hirano , M. Shirobe , and K. Iijima , “Oral Frailty and the Trajectories of Psychological Well‐Being and Cognitive Function: Findings From the 12‐Year Community‐Based Kashiwa Study,” Journals of Gerontology. Series A, Biological Sciences and Medical Sciences 81 (2026): glag006. 41533683 10.1093/gerona/glag006

[16] S. Khairinisa , S. Kiuchi , Y. Matsuyama , M. Iwasaki , and J. Aida , “Oral Frailty, Dental Visits, and Healthy Life Expectancy: A 6‐Year Prospective Cohort Among Japanese Older Adults,” Geriatrics & Gerontology International 25, no. 12 (2025): 1884–1893. 41117508 10.1111/ggi.70230 PMC12719132

[17] K. Eaton , H. Yusuf , and P. Vassallo , “Editorial: The WHO Global Oral Health Action Plan 2023‐2030,” Community Dental Health 40, no. 2 (2023): 68–69. 37265395 10.1922/CDH_Jun23Editorial02

[18] Federation FWD , “FDI POLICY STATEMENT Oral Health for Healthy Ageing,” https://www.fdiworlddental.org/sites/default/files/2023‐10/8.%20EN_FDPS3_Oral%20Health%20for%20Healthy%20Ageing.pdf.

[19] Federation FDIWD , “FDI Policy Statement on Oral Health for Healthy Ageing: Adopted by the FDI General Assembly: 24 September 2015, Bangkok, Thailand,” International Dental Journal 66, no. 1 (2016): 7–8. 26803941 10.1111/idj.12231 PMC9376517

[20] K. G. H. Parisius , M. C. Verhoeff , F. Lobbezoo , et al., “Towards an Operational Definition of Oral Frailty: A e‐Delphi Study,” Archives of Gerontology and Geriatrics 117 (2024): 105181. 37713933 10.1016/j.archger.2023.105181

[21] H. L. Huang , K. Matsuo , S. T. Huang , et al., “Chinese Version of the Oral Frailty Five‐Item Checklist,” Geriatrics & Gerontology International 25, no. 5 (2025): 709–710. 40129217 10.1111/ggi.70032

[22] Y. Zhou , L. Zhou , W. Zhang , et al., “Prevalence and Influencing Factors of Oral Frailty in Older Adults: A Systematic Review and Meta‐Analysis,” Frontiers in Public Health 12 (2024): 1457187. 39735756 10.3389/fpubh.2024.1457187 PMC11671401

[23] T. Tanaka , W. Lyu , Y. Yoshizawa , B. K. Son , and K. Iijima , “Predictive Validity of Senior Volunteer‐Led Frailty Check‐Up Results for Disability and Mortality Among Community‐Dwelling Older Adults: A Cohort Study,” Archives of Gerontology and Geriatrics 139 (2025): 105998. 40916323 10.1016/j.archger.2025.105998

[24] T. Tanaka , W. Lyu , and K. Iijima , “Community Well‐Being and Functional Disability Risk Among Older Adults: A Multilevel Analysis of 91 Municipalities in Japan,” Geriatrics & Gerontology International 26, no. 1 (2026): e70337. 41562137 10.1111/ggi.70337 PMC12820745

[25] S. Satake and H. Arai , “Questionnaire for Medical Checkup of Old‐Old (QMCOO),” Geriatrics & Gerontology International 20, no. 10 (2020): 991–992. 33003256 10.1111/ggi.14004

[26] T. Tanaka , Y. Yoshizawa , K. Sugaya , et al., “Predictive Validity of the Questionnaire for Medical Checkup of Old‐Old for Functional Disability: Using the National Health Insurance Database System,” Geriatrics & Gerontology International 23, no. 2 (2023): 124–130. 36639356 10.1111/ggi.14533

[27] T. Tanaka , Y. Yoshizawa , K. Kitamura , et al., “Frailty Determined by the Questionnaire for Medical Checkup of Old‐Old Is Correlated With Increased Healthcare Cost: Using the Japanese Health Insurance Database System,” Geriatrics & Gerontology International 23, no. 12 (2023): 973–974. 37855182 10.1111/ggi.14711 PMC11503604

[28] H. Makizako , D. Shiratsuchi , S. Akaida , et al., “Effects of Digital‐Based Interventions on the Outcomes of the Eligibility Criteria for Sarcopenia in Healthy Older Adults: A Systematic Review and Meta‐Analysis,” Ageing Research Reviews 104 (2025): 102663. 39814237 10.1016/j.arr.2025.102663

[29] Gerontology NCGG , “Guidelines for Digital Health in the Prevention and Improvement of Sarcopenia and Frailty,” Minds Guideline Registry, Japan Council for Quality Health Care, 2025, https://healthcare‐service.amed.go.jp/assets24/pdf/guidelines_healthcare_services_SF.pdf.

